# Progranulin Improves Acute Lung Injury through Regulating the Differentiation of Regulatory T Cells and Interleukin-10 Immunomodulation to Promote Macrophage Polarization

**DOI:** 10.1155/2020/9704327

**Published:** 2020-05-31

**Authors:** Yan-qing Chen, Chuan-jiang Wang, Ke Xie, Ming Lei, Yu-sen Chai, Fang Xu, Shi-hui Lin

**Affiliations:** ^1^Department of Critical Care Medicine, The First Affiliated Hospital of Chongqing Medical University, Chongqing, China; ^2^Department of Critical Care Medicine, The Seventh Affiliated Hospital, Sun Yat-sen University, Shenzhen, China

## Abstract

Progranulin (PGRN), which plays an anti-inflammatory role in acute lung injury (ALI), is promising as a potential drug. Studies have shown that regulatory T cells (Tregs) and interleukin- (IL-) 10 can repress inflammation and alleviate tissue damage during ALI. In this study, we built a lipopolysaccharide- (LPS-) induced ALI mouse model to illustrate the effect of PGRN on regulation of Treg differentiation and modulation of IL-10 promoting macrophage polarization. We found that the proportion of Tregs in splenic mononuclear cells and peripheral blood mononuclear cells was higher after treatment with PGRN. The increased proportion of Tregs after PGRN intratracheal instillation was consistent with the decreased severity of lung injury, the reduction of proinflammatory cytokines, and the increase of anti-inflammatory cytokines. *In vitro*, the percentages of CD4^+^CD25^+^FOXP3^+^ Tregs from splenic naïve CD4^+^ T cells increased after PGRN treatment. In further research, it was found that PGRN can regulate the anti-inflammatory factor IL-10 and affect the polarization of M1/M2 macrophages by upregulating IL-10. These findings show that PGRN likely plays a protective role in ALI by promoting Treg differentiation and activating IL-10 immunomodulation.

## 1. Introduction

Acute respiratory distress syndrome (ARDS) is an acute lung injury (ALI) [1] first described in 1967 [2] that is characterized by clinical hypoxemia and morphologically by diffuse alveolar damage [1]. In a study of 459 intensive care units (ICUs) from 50 countries across 5 continents, the incidence of ARDS in the ICUs was 10.4%, and the mortality was more than 34.9% [3]. Even with the increased recognition of ARDS, its identification still depends on various clinical and imaging signs [4], and the treatment of ARDS relies on supportive therapy [5]. Lipopolysaccharide (LPS), a part of the outer membrane of gram-negative bacteria, is one of the most important causes of ALI [6, 7]. Intratracheal administration of LPS in mice leads to edema caused by alveolar inflammation, which simulates ARDS in experimental animal model, visually [8]. Therefore, we used LPS-induced ALI mouse models [7, 9–11] to explore specific treatments that are based on potential biomarkers.

Progranulin (PGRN) is widely expressed in epithelia, immune cells, and neurons [12] and is involved in diverse physiological functions and disease processes, including wound healing, host defence, and neurodegeneration [13–15]. As a mediator of inflammation [16], although its anti-inflammatory role is widely accepted, the exact function of PGRN may be distinct because of the diverse stages and components of inflammation [17]. Because of its higher affinity for TNF receptors (especially TNFR2) than TNF-*α*, PGRN exerts an anti-inflammatory effect and inhibits TNF-*α* signalling [18]. A few studies have shown that PGRN ameliorates lipopolysaccharide- (LPS-) induced lung injury through PGRN/TNFR2 interaction [19] and is expressed by human and mouse CD4^+^Foxp3^+^ regulatory T cells (Tregs) rather than TNFR1 [20]. Moreover, the coexpression of CD25 best highlights the suppression capacity of the Treg population [21]. On the one hand, TNF-*α* promotes the proliferation and expansion of Tregs; on the other hand, it can downregulate the suppression capacity of Tregs, exerting both anti-inflammatory and proinflammatory roles. Tregs can secrete interleukin- (IL-) 10, an anti-inflammatory cytokine, to suppress hypernomic immune responses [22, 23]. In this way, ALI may be ameliorated by restraining the production of TNF-*α* and neutrophil activity [24]. Furthermore, promoting the differentiation of Tregs from CD4^+^ naïve T cells *in vitro* and increasing the production of IL-10 also mediate the anti-inflammatory role of PGRN [25, 26]. As a consequence, two questions stand out: (1) does the protective effect of PGRN involve the regulation of Tregs and IL-10 immune modulation in ALI? (2) If so, does the expression of IL-10 regulated by PGRN stem from CD4^+^ naïve T cells?

Here, we establish an LPS-induced ALI mouse model, measured the percentage of Tregs in splenic mononuclear cells (MNCs) and peripheral blood mononuclear cells (PBMCs), the polarization of macrophages in lung tissue, and the plasma levels of cytokine/chemokine. In addition, we cultured CD4^+^ naïve T cells and RAW 264.7 cells to illuminate the role of PGRN in Treg differentiation and macrophage polarization.

## 2. Materials and Methods

### 2.1. Animals

C57/BL6 mice (6-8 weeks) were purchased from Chongqing Medical University. Progranulin-deficient (PGRN^−/−^) mice with a C57/BL6 background were purchased from the Jackson Laboratory and maintained at Chongqing Medical University. This study was approved by the Ethics Committees of the First Affiliated Hospital of Chongqing Medical University (2016–34). All animal experiments were conducted in accordance with the Institutional Animal Care and Use Committee of Chongqing Medical University.

### 2.2. LPS-Induced ALI Mouse Model and PGRN Treatment

LPS-induced ALI was performed to establish an ALI mouse model. Briefly, 1 mg/mL of LPS (Escherichia coli, serotype 055:B5; Sigma-Aldrich, St. Louis, MO, USA) was injected into mice through intratracheal instillation, and the control group was injected with the same volume of sterile phosphate-buffered saline (PBS), as in Wang et al. [27]. Mice were then sacrificed under ether narcotization at 24 h after challenge with LPS or PBS to collect 1% heparin-anticoagulated peripheral whole blood, spleen, bronchoalveolar lavage fluid (BALF), and lung tissues. The WT+LPS+PGRN and PGRN^−/−^+LPS+PGRN groups were treated with 2 *μ*g of recombinant-mouse PGRN protein (CF, R&D Systems, Minneapolis, MN, USA) 30 min after LPS challenge through intratracheal instillation [19]. Blood, spleen, BALF, and lung tissues were collected as indicated for further analysis.

### 2.3. Lung W/D Weight Ratio

Lung W/D weight ratios were determined to reflect pulmonary edema. The right lung of each mouse was weighed immediately after collection as the wet weight. Then, the lung was reweighed after being dried in an oven at 60°C for 48 hours as the dry weight. The W/D ratio was calculated as the wet weight divided by the dry weight [28].

### 2.4. Histopathology

Lung tissues were fixed, embedded, sectioned, and stained with haematoxylin and eosin (H&E) for histopathology analysis. The lung injury scores were estimated in a blinded manner based on the pathological ALI scoring system from the American Thoracic Association [29], which comprises the following four categories of lung injury: alveolar congestion, hemorrhage, gap or vascular wall neutrophil infiltration or aggregation, and alveolar septal thickening or transparent membrane formation with a score of 0 to 4 in each domain as follows: 0: no or very slight damage, 1: mild injury, 2: moderate injury, 3: severe injury, and 4: very severe damage. The total score of the cumulative lesions was taken as the pathological score of the ALI. The higher the score was, the more severe the injury.

### 2.5. Terminal Deoxynucleotidyl Transferase-Mediated dUTP Nick End Labelling (TUNEL) Staining

In Situ Cell Death Detection Kit (Roche, Switzerland) was applied to detect the apoptosis rate in lung tissue sections according to the manufacturer's instruction. Each section in 5 random fields under ×400 magnification was shot, and the percentage of TUNEL-positive cells was calculated.

### 2.6. Immunohistochemistry

Xylene was used to dewax the lung sections, which were then put through a gradient alcohol series for rehydration. After antigen retrieval, 3% H_2_O_2_ was used to block the endogenous peroxidase activity for 25 min in the dark. Subsequently, the sections were incubated in normal goat serum for 30 min at room temperature. Rabbit anti-mouse IL-10 polyclonal antibody (1 : 200, Servicebio, Hubei, China) or rabbit anti-mouse myeloperoxidase (MPO) polyclonal antibody (1 : 200, Servicebio) was then added to the sections, and incubation was performed overnight at 4°C in a humidified chamber. The sections were washed, and a streptavidin-peroxidase complex was added (Zhongshan, Beijing, China). Diaminobenzidine was used to allow the IL-10 or MPO to be visualized, and haematoxylin was used to dye the cell nuclei. Dehydration with a gradient alcohol series was performed, and the sections were placed in xylene for 10 min for differentiation, after which point neutral gum sealing of the pieces was performed. Images were obtained using a LEICA CTR 5500 (Leica Camera, Wetzlar, Germany) and were analysed with Image-pro Plus 6.0 software.

### 2.7. Mouse Plasma Cytokine/Chemokine

Blood was collected from mice as previously described and then centrifuged at 3000 rpm for 15 min at 4°C to obtain supernatant plasma. IL-1*β*, IL-6, IL-10, IL-17A, TNF-*α*, and CXCL1 were determined with a Mouse Cytokine/Chemokine Magnetic Bead Panel Kit (eBioscience, San Diego, CA, USA).

### 2.8. Polymorphonuclear Cell Counts in BALF

BALF was collected after instilling into mouse lungs with 1 mL of sterile PBS at 4°C for three times as described previously [30]. BALF was centrifuged at 1500g for 10 min at 4°C and resuspended in PBS for cytospin preparation. Total neutrophil numbers in BALF were counted after Wright-Giemsa staining.

### 2.9. PBMCs and Splenic Mononuclear Cell Isolation

PBMCs were fractionated from mouse heparin-anticoagulated whole blood using a mouse mononuclear cell isolation kit (TBD Science, Tianjin, China) according to the manufacturer's instructions. Splenic mononuclear cells were isolated from splenic cell suspensions by a 60% Percoll Plus density gradient (1.077 g/mL, GE Healthcare, Chicago, IL, USA).

### 2.10. Immunofluorescence

Lung paraffin sections were deparaffinized with xylene and rehydrated in a graded series of alcohol. Tris-ethylene diamine tetraacetic acid (EDTA) retrieval solution (PH 8.0, Servicebio) was used for microwave antigen retrieval, and then, normal goat serum was used to incubate the sections. Rabbit anti-mouse F4/80 polyclonal antibody (1 : 5000, Servicebio), rabbit anti-mouse iNOS polyclonal antibody (1 : 5000, Servicebio), and rabbit anti-mouse CD206 polyclonal antibody (1 : 200, Servicebio) were added to the sections, and incubation was performed overnight at 4°C in a humidified chamber. The sections were subsequently incubated with goat anti-rabbit antibody conjugated with fluorescein isothiocyanate (FITC) for F4/80, Cy3 for iNOS, and Cy5 for CD206 at room temperature in the dark. 4′,6-Diamidino-2-phenylindole (DAPI) was used to dye the cell nuclei. Neutral gum sealing was performed, and images were obtained using a LEICA CTR 5500 (Leica Camera) and analysed with Image-pro Plus 6.0 software.

### 2.11. Flow Cytometry

Blank controls of cultured T cells, isolated PBMCs, and splenic MNCs were treated the same as samples, except for incubation with fluorescent antibody. Isotype controls of isolated PBMCs and splenic MNCs were treated the same as blank controls but were incubated with rat IgG2a kappa isotype control (Ebr2a) and allophycocyanin (APC) (eBioscience) after fixation/permeabilization. Cultured T cells, isolated PBMCs, and splenic mononuclear cells were incubated with fluorescent antibody to test the percentages of Tregs (CD4^+^CD25^+^Foxp3^+^). To stain CD4, CD25, and Foxp3, anti-CD4-FITC, anti-CD25-phycoerythrin (PE), anti-Foxp3-APC, and a Fixation/Permeabilization kit (eBioscience) were used according to the manufacturer's instructions. At least 10^5^ cells were collected with a CytoFLEX flow cytometer (Beckman Coulter, Pasadena, CA, USA) and were analysed with FlowJo software V10.

### 2.12. Naïve CD4^+^ T Cell Isolation and Differentiation into Tregs *In Vitro*

The collection of the spleen from C57BL/6 mice is described elsewhere [31]. Cell pellets were resuspended in PBS+ containing fluorescent antibodies directed against CD62L, CD44, CD25, and CD4 to separate the naïve CD4^+^T cells. Approximately 5 × 10^5^ naïve CD4^+^ T cells were plated in 48-well plates in 0.5 mL of complete RPMI 1640 medium (Gibco, Grand Island, USA) with or without 200 ng/mL PGRN and were incubated for 3 days at 37°C with 5% CO_2_ prior to flow cytometry (CD4-FITC, CD25-PE, or FOXP3-APC) (*n* = 5). Naïve CD4^+^ T cells were stimulated with coated anti-mouse CD3 (5 *μ*g/mL, eBioscience) and anti-mouse CD28 (2 *μ*g/mL, eBioscience). The culture medium was supplemented with the cytokines IL-2 (10 ng/mL, PeproTech, Rocky Hill, NJ, USA) and transforming growth factor- (TGF-) *β*1 (50 ng/mL, PeproTech), 2 mM L-glutamine (STEMCELL Technologies, Vancouver, Canada), and 50 mM *β*-mercaptoethanol (Macklin, Shanghai, China) as described previously [32].

### 2.13. RAW 264.7 Macrophage Culture and Treatment

RAW 264.7 macrophages were obtained from the American Type Culture Collection (ATCC, Rockville, MD, USA), aliquoted and frozen in Fibulas's BioFlash Drive SP One Step Controlled-Rate Freezing Kit (Fibulas, New York, USA), and then thawed and cultured in Dulbecco's modified Eagle's medium (DMEM; Gibco) supplemented with 10% heat-inactivated foetal bovine serum (FBS; Gibco). The cells were incubated in a humidified atmosphere at 37°C with 5% CO_2_. The RAW 264.7 macrophages were treated as follows. (1) Cells incubated with culture medium were designated as the control group. (2) Cells stimulated with LPS (30 ng/mL; Sigma-Aldrich) were designated as the LPS group. (3) Cells stimulated with LPS (30 ng/mL; Sigma-Aldrich) plus IL-10 (100 ng/mL; R&D Systems, Minneapolis, USA) were designated as the LPS+IL-10 group. (4) Cells stimulated with LPS (30 ng/mL) plus PGRN (500 ng/mL) [33] were designated as the LPS+PGRN group. (5) Cells stimulated with LPS (30 ng/mL) plus IL-10 (100 ng/mL) and PGRN (500 ng/mL) were designated as the LPS+IL-10+PGRN group. After 24 h, the membrane surface molecules were stained with 3 *μ*L/test of PE-conjugated anti-mouse CD86 monoclonal antibody (Mab; Invitrogen, California, USA) or APC-conjugated anti-mouse CD206 Mab (Invitrogen, California, USA) for 30 min at room temperature in the dark as per the manufacturer's instructions. The mean fluorescence intensity of the cell surface molecules was assessed by flow cytometry.

### 2.14. Statistical Analyses

Statistical analyses were performed with GraphPad Prism 6 (GraphPad Software, San Diego, CA, USA) and SPSS 20.0 (IBM, Armonk, NY, USA). Data are reported as the means ± standard deviation (SD). Differences between groups were assessed by one-way analysis of variance (ANOVA) followed by the Tukey Post Hoc test. *p* < 0.05 was considered to be significantly different.

## 3. Results

### 3.1. PGRN Alleviated Lung Injury in LPS-Induced ALI Mice

**Figure 1:**
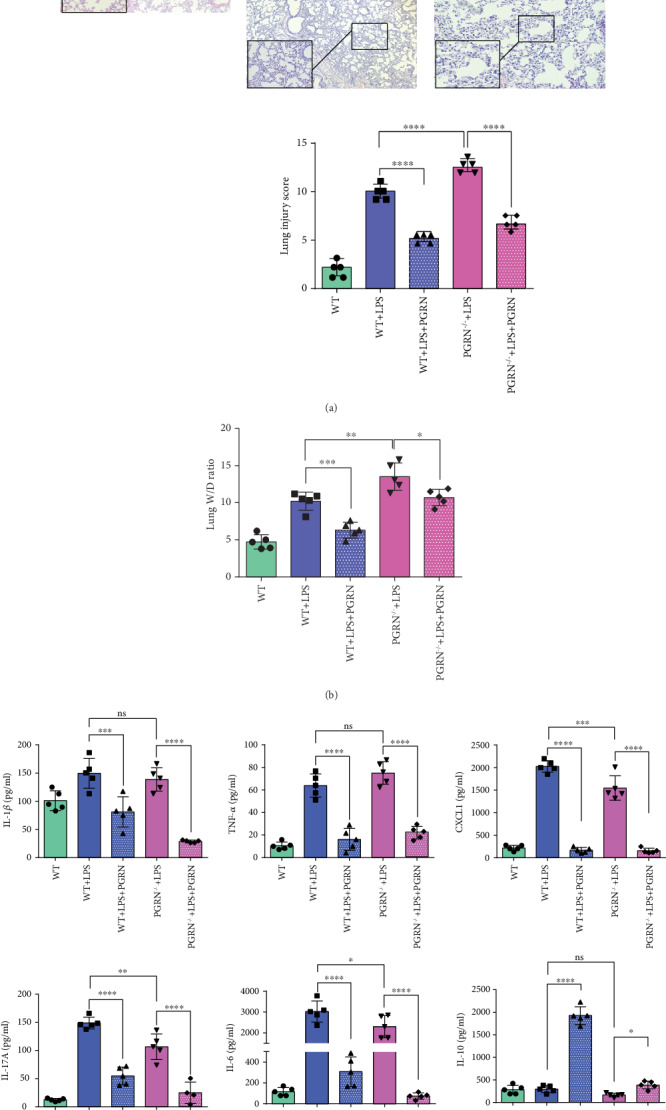
PGRN has an anti-inflammatory role in LPS-induced ALI. C57BL/6 mice were randomly divided into WT, WT+LPS, WT+LPS+PGRN, and PGRN-deficient (PGRN^−/−^) mice with a C57/BL6 background which were randomly divided into the PGRN^−/−^+LPS and PGRN^−/−^+LPS+PGRN groups (*n* = 5/group). (a) The lungs from each experimental group were processed for histological examination after H&E staining. Compared with the WT group, thickened alveolar wall, alveolar hemorrhage and collapse, and inflammatory cell in filtration were less severe and were treated with PGRN 30 min after LPS challenge. The trend was the same in the PGRN^−/−^ group. (b) PGRN alleviates pulmonary edema in the LPS-induced mouse model. Pulmonary edema was measured by lung W/D weight ratio. (c) PGRN plays a potential anti-inflammatory role in the LPS-induced ALI mouse model. IL-1*β*, IL-6, IL-10, IL-17A, TNF-*α*, and CXCL1 expression levels in sera of mice were detected using a Mouse Cytokine/Chemokine Magnetic Bead Panel Kit. PGRN downregulated the production of proinflammatory cytokines/chemokines in plasma, including IL-1*β*, IL-6, IL-17A, TNF-*α*, and CXCL1, and upregulated the expression of the anti-inflammatory cytokine IL-10 in plasma in all experimental groups. ns: not significant; ∗*p* < 0.05, ∗∗*p* < 0.01, ∗∗∗*p* < 0.001, and ∗∗∗∗*p* < 0.0001 by the one-way ANOVA followed by the Tukey Post Hoc test comparing the WT, WT+LPS, WT+LPS+PGRN, PGRN^−/−^+LPS, and PGRN^−/−^+LPS+PGRN groups.

**Figure 2:**
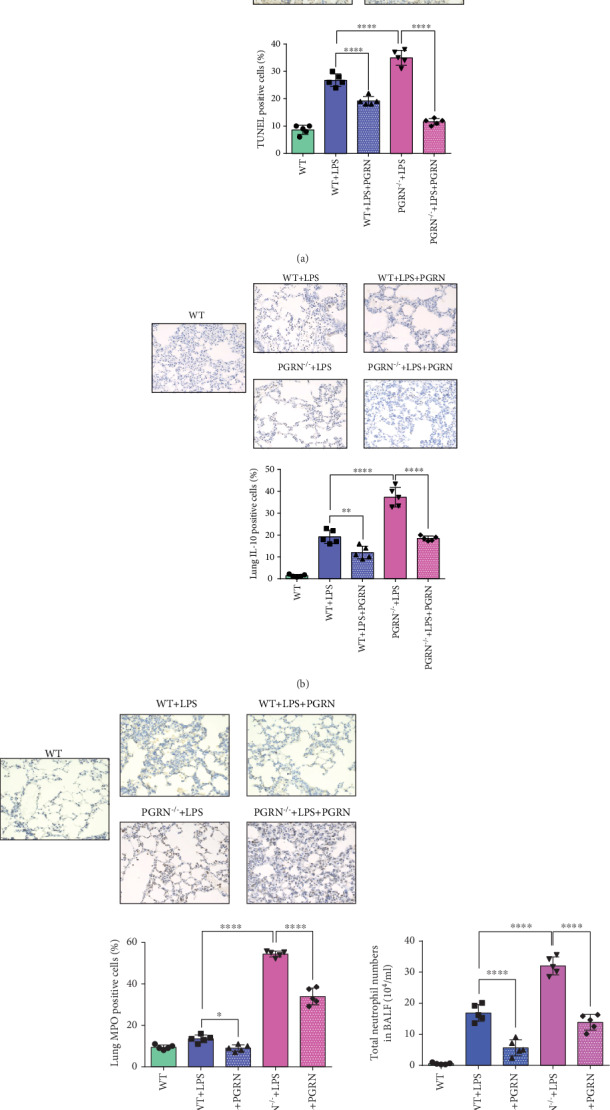
PGRN ameliorated LPS-induced ALI through antiapoptosis, inflammatory infiltration, and neutrophil aggregation. C57BL/6 mice were randomly divided into WT, WT+LPS, WT+LPS+PGRN, and PGRN-deficient (PGRN^−/−^) mice with a C57/BL6 background which were randomly divided into the PGRN^−/−^+LPS and PGRN^−/−^+LPS+PGRN groups (*n* = 5/group). (a) PGRN had an antiapoptotic effect on the lungs in the LPS-induced ALI mouse model. Apoptosis was detected by TUNEL staining, and strongly positive apoptosis appeared in the lungs of the WT+LPS and PGRN^−/−^+LPS groups compared with the WT group. Apoptosis was reduced in mice treated with PGRN. (b) PGRN exerted a protective effect on the LPS-induced ALI mouse model, at least in part by IL-10 immune modulation. The expression of IL-10 in the lung tissues increased significantly in the WT+LPS and PGRN^−/−^+LPS groups compared to the WT group. After the intervention of PGRN, IL-10 expression declined significantly. (c) PGRN reduced the level of MPO-producing neutrophils and played a protective role in the LPS-induced ALI mouse model. (d) PGRN reduced the total number of PMNs in BALF in the LPS-induced mouse models. ns: not significant; ∗∗*p* < 0.01and ∗∗∗∗*p* < 0.0001 by one-way ANOVA followed by the Tukey Post Hoc test comparing the WT, WT+LPS, WT+LPS+PGRN, PGRN^−/−^+LPS, and PGRN^−/−^+LPS+PGRN groups. Representative data are shown.

To evaluate the protective effect of PGRN in our LPS-induced ALI mouse model, we measured the lung injury from each experimental group through histological examination after H&E staining. Compared with the WT group, the LPS-induced ALI in the WT+LPS and PGRN^−/−^+LPS groups had higher lung injury scores, with alveolar congestion, hemorrhage, vascular wall neutrophil infiltration or aggregation, alveolar septal thickening, and transparent membrane formation. After treatment with PGRN, the lung injury scores were both significantly reduced compared with those in their corresponding LPS-induced ALI groups (*p* < 0.0001; Figure 1(a)). Subsequently, infiltration of neutrophils and macrophages was confirmed with immunohistochemistry (IHC) of MPO in the lungs (Figure 2(c)). Infiltration of neutrophils and macrophages in the WT+LPS group and the PGRN^−/−^+LPS group increased, compared with that in the WT group. And PGRN treatment relieved the damage from the infiltration of neutrophils and macrophages in WT (*p* < 0.05) and PGRN^−/−^ (*p* < 0.0001) mice, respectively. Moreover, pulmonary edema is a hallmark of ALI/ARDS; we determined lung W/D weight ratio as an indicator of pulmonary edema. Consistent with lung injury scores, the lung W/D weight ratios of the WT+LPS group and the PGRN^−/−^+LPS group were higher than those of the WT group. After intratracheal instillation with PGRN, the lung W/D weight ratios were reduced in WT (*p* < 0.001) mice and PGRN^−/−^ (*p* < 0.05) mice, respectively (Figure 1(b)), which means pulmonary edema reduced. In addition, the lung injury mentioned above of the PGRN^−/−^+LPS group was more severe than that of the WT+LPS group.

### 3.2. PGRN Had an Antiapoptotic Effect on the Lung in an LPS-Induced ALI Mouse Model

Considering that apoptosis of alveolar epithelia is a characteristic of ALI, we estimated apoptosis in lung tissues with TUNEL assays (Figure 2(a)). The pulmonary apoptosis of the PGRN^−/−^+LPS group was more severe than that of the WT+LPS group (*p* < 0.0001). After administration of recombinant PGRN protein, the apoptosis of alveolar epithelial cells of the WT+LPS+PGRN group was significantly lower than that of the WT+LPS group (*p* < 0.0001). In addition, we observed the same phenomenon in the PGRN^−/−^ group (*p* < 0.0001).

### 3.3. PGRN Restrained the Inflammation in the LPS-Induced ALI Mouse Model

To further clarify the anti-inflammatory role of PGRN in the LPS-induced ALI mouse model, we determined the cytokine/chemokine levels in plasma (Figure 1(c)) and the total neutrophil numbers in BALF. We found that all cytokine/chemokine levels increased in the WT and PGRN^−/−^ mice after LPS challenge. After treatment with PGRN, all cytokine/chemokine levels decreased, except for IL-10. PGRN treatment increased the plasma levels of the anti-inflammatory cytokine IL-10 in both the WT+LPS+PGRN (*p* < 0.0001) and PGRN^−/−^+LPS+PGRN groups (*p* < 0.05), compared to the WT+LPS group and the PGRN^−/−^+LPS group, respectively. Intriguingly, contrary to the plasma level of IL-10, the expression of IL-10 in lung tissues of IHC analysis (Figure 2(b)) decreased after PGRN instillation in WT mice (*p* < 0.01) and PGRN^−/−^ mice (*p* < 0.0001). In addition, polymorphonuclear cells (PMNs) are the first responders to the inflammatory microenvironment, and the number of PMNs in the pulmonary pool changes to pulmonary inflammation. We counted the total neutrophil numbers in BALF (Figure 2(d)) and found that PMNs dramatically increased in WT mice after challenge with LPS. With treatment with PGRN, compared to the WT+LPS group, the PMNs of the WT+LPS+PGRN group decreased significantly (*p* < 0.0001). In PGRN^−/−^ mice, the PMN numbers of the PGRN^−/−^+LPS group were higher than those of the PGRN^−/−^+LPS+PGRN group (*p* < 0.0001), similar to WT mice.

### 3.4. PGRN Augmented the Proportions of Tregs in Splenic MNCs and PBMCs of the LPS-Induced ALI Mouse Model

**Figure 3:**
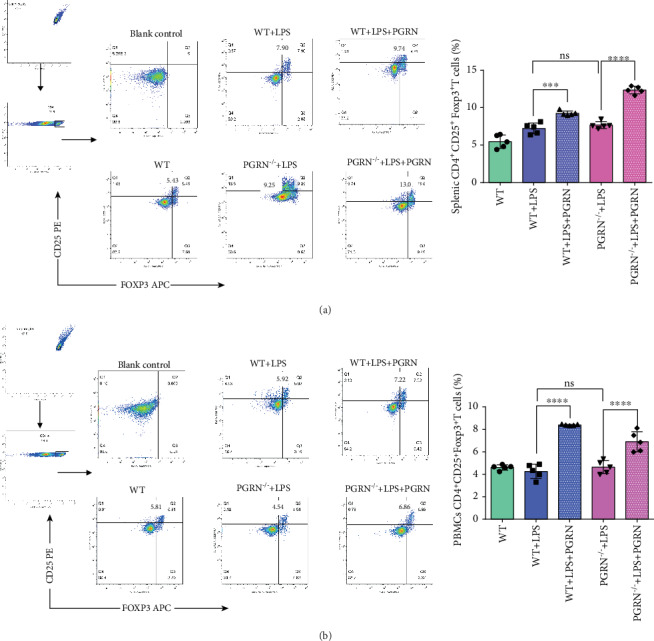
PGRN treatment increased CD4^+^CD25^+^Foxp3^+^ Treg proportions significantly in PBMCs and splenic MNCs of the WT+LPS and PGRN^−/−^+LPS groups. (a, b) CD4^+^CD25^+^Foxp3^+^ Tregs in PBMCs and splenic MNCs were detected by flow cytometry, and the proportions were analysed using FlowJo. ns: not significant; ∗∗∗*p* < 0.001 and ∗∗∗∗*p* < 0.0001 by one-way ANOVA followed by Tukey Post Hoc test comparing the WT, WT+LPS, WT+LPS+PGRN, PGRN^−/−^+LPS, and PGRN^−/−^+LPS+PGRN groups. In each group, *n* = 5; three replicate experiments were performed three times, and the results were in good agreement. Representative data are shown.

Furthermore, because PGRN can promote the proliferation of Treg cells and improve the anti-inflammatory and immunosuppressive effects, we used a flow cytometric method to determine if PGRN induces Treg cell differentiation in blood and spleen involved in LPS-induced ALI models. The outcomes showed that Tregs in splenic MNCs and PBMCs displayed similar tendencies. The Treg levels in both cell types increased after treatment with PGRN in both the WT and PGRN^−/−^ mice, although the range of increase differed (Figures 3(a) and 3(b)).

### 3.5. M1 and M2 Macrophage Phenotypes in the Lung Are Regulated by PGRN

**Figure 4:**
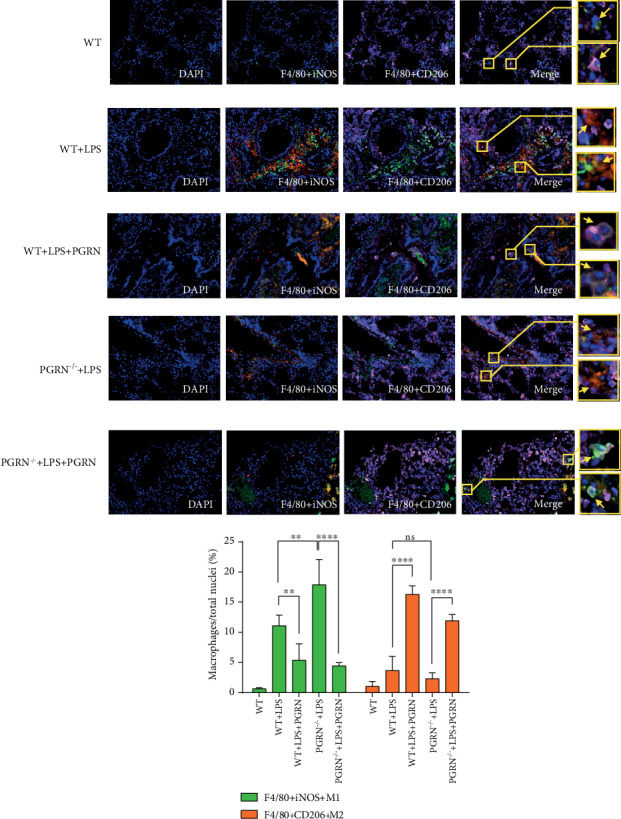
PGRN treatment reduced M1 macrophage proportions but increased M2 macrophage proportions in the lungs of the LPS-induced ALI mouse model and showed a protective role in ALI. Macrophages were fixed and immunolabelled with a pan-macrophage marker (F4/80: green fluorescence) and strong indicators of M1 (iNOS: red fluorescence) and M2 (CD206: pink fluorescence) phenotypes. Five 400-fold fields of positive cells were counted and analysed. ns: not significant; ∗∗*p* < 0.01 and ∗∗∗∗*p* < 0.0001 by one-way ANOVA followed by the Tukey Post Hoc test comparing the WT, WT+LPS, WT+LPS+PGRN, PGRN^−/−^+LPS, and PGRN^−/−^+LPS+PGRN groups. In each group, *n* = 5; three replicate experiments were performed three times, and the results were in good agreement. Representative data are shown.

Macrophages play important roles in inflammation and repair in the lungs and alveolar spaces. We used immunofluorescence to assess the effect of PGRN on macrophages. First, we discovered that both M1 and M2 macrophage phenotypes increased in the WT+LPS and PGRN^−/−^+LPS groups compared with the WT group. Meanwhile, compared with the WT+LPS group, M1 macrophage phenotypes increased more in the PGRN^−/−^+LPS group, while the trend for M2 macrophages was opposite. Eventually, PGRN treatment can significantly reduce the proportion of M1 macrophages and increase the phenotypes of M2 macrophages in the WT+LPS+PGRN group and the PGRN^−/−^+LPS+PGRN group (Figure 4).

### 3.6. *In Vitro*, PGRN Promotes Treg Differentiation from Naive CD4^+^ T Cells and Improves the Polarization of M1/M2 Macrophages

**Figure 5:**
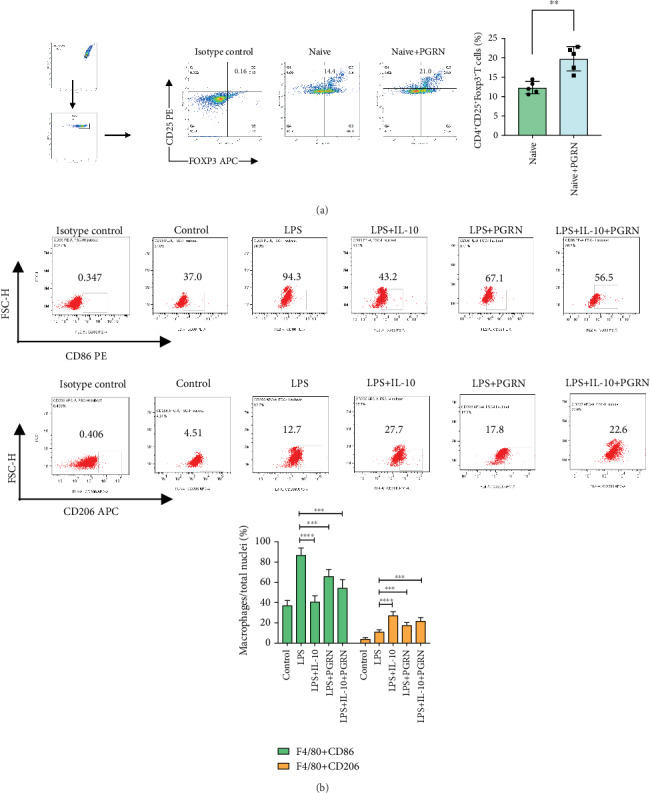
PGRN promotes Treg differentiation of naive CD4^+^ T cells *in vitro* and improves the polarization of M1/M2 macrophages. (a) PGRN significantly promotes the differentiation of CD4^+^CD25^+^Foxp3^+^ Tregs from CD4^+^ naïve T cells. CD4^+^ naïve T cells were sorted from the spleens of wild-type C57BL/6 mice and cultured. Tregs were detected by flow cytometry on day 3 and analysed by FlowJo. ∗∗*p* < 0.01 by Student's *t* test comparing controls and the PGRN intervention group. (b) PGRN and IL-10 can affect the polarization of M1/M2 macrophages. M1/M2 types were examined by flow cytometry on day 3 and analysed by FlowJo. ∗∗∗∗*p* < 0.0001 by one-way ANOVA followed by the Tukey Post Hoc test comparing the control, LPS, LPS+IL-10, LPS+PGRN, and LPS+IL-10+PGRN groups. In each group, *n* = 5; three replicate experiments were performed three times, and the results were in good agreement. Representative data are shown.

To confirm whether PGRN can affect naïve CD4^+^ T lymphocyte differentiation into Treg cells, we extracted naïve CD4^+^ T lymphocytes from the spleens of mice for *in vitro* cell culture. Next, recombinant PGRN was added to the medium for intervention. After 3 days of training, naïve CD4^+^ T lymphocytes differentiated more Treg cells after treatment with recombinant PGRN (Figure 5(a)). Our animal experiments found that PGRN can regulate IL-10 expression in plasma and lung tissue. Further, *in vitro* cell experiments confirmed that PGRN and the anti-inflammatory cytokine IL-10 can promote the polarization of M2 macrophages and reduce the polarization of M1 macrophages, separately (Figure 5(b)).

## 4. Discussion

ARDS is a heterogeneous syndrome [1], not only between patients but also in the same patient, with diverse causes and stages of the same disease, which makes it difficult to understand its mechanism independently or to treat it programmatically [34]. Despite 50 years of study, there is no specific pharmacological therapy for ARDS. Uncontrolled inflammation is currently recognized as the core issue of ARDS. Numerous inflammatory mediators are produced, and many inflammatory cells participate in the inflammatory injury process [35] during ARDS. Biotherapies, including micro ribonucleic acid (miRNA), mesenchymal stem cells, and food and drug administration- (FDA-) approved IL-10 [36, 37], are among the current therapeutic modalities for ARDS. Biotherapeutics have been shown to be an important means of controlling inflammation in this syndrome and show promise for the further development of treatment strategies.

PGRN, a secreted glycoprotein, is abundantly expressed in a broad range of tissues and cell types with pleiotropic functions [38]. PGRN can act as a universal regulator of cell growth, migration and transformation, cell cycle, wound healing, tumourigenesis, and cytotoxic drug resistance as a secreted glycoprotein. In autoimmune diseases, its anti-inflammatory role and therapeutic application have been broadly explored [18, 39–44]. PGRN overexpression can induce the secretion of some inflammatory cytokines (e.g., IL-8, IL-6, IL-10, and TNF-*α*). Thus, the anti-inflammatory properties of PGRN highlight its potential for novel therapeutic use in inflammatory diseases. Our research team previously confirmed that PGRN-mediated protection against sepsis is closely linked to improved peritoneal macrophage recruitment [45]. In ALI, a few studies have shown a protective role for PGRN [10, 19]. In one of them, 800 *μ*g of LPS (E. coli 055:B5; Sigma) was intratracheal instillated to female BALB/c mice to induce an ALI animal model. It demonstrated that PGRN could effectively ameliorate the LPS-induced ALI in mice [19]. We reconstructed the model and explored the possible mechanism. Intratracheal instillation of PGRN 30 min after LPS challenge is a posttreatment. But, there is no sufficient evidence that the lung injury occurred within a short period (such as 30 min) after LPS challenge. Therefore, it is incomplete to describe the therapeutic effect of PGRN. The time point of the formation of lung injury in animal models remains further explored and figured out. It is the key to discussion on the occurrence and treatment of lung injury at early phase. In the other one, 1 mg/kg or 25 mg/kg of LPS (Escherichia coli O55:B5, Sigma, St. Louis, MO) was used in male C57BL/6 mice via intratracheal instillation to induce ALI and generate the ALI model. This study shows that PGRN-targeting microRNA miR-34b-5p inhibition attenuates lung inflammation and apoptosis in an LPS-induced acute lung injury mouse model [10]. To make the therapeutic point of PGRN, blocking and adding PGRN in WT after the injury was induced can give us strong evidence. But PGRN might mediate its anti-inflammatory effects, at least in part, by blocking TNF-*α* binding to its receptors. And TNFR2 seemed to play an important role in ARDS [19]. Coupled with the role of endogenous PGRN, it is difficult to completely block the source of PGRN. Therefore, we selected the treatment of PGRN^−/−^ mice to verify the effect of PGRN deficiency on ALI and explore the potential therapeutic effect initially. In this study, we found that compared to WT mice, the PGRN^−/−^ mice were more susceptive to LPS-induced acute lung injury, including pulmonary neutrophil infiltration or aggregation, edema, apoptosis, and inflammatory microenvironment (Figures 1, 2, and 4). That was consistent with the perspective that mice lacking endogenous PGRN would develop exaggerated inflammatory tissue damage when they encountered stimuli [46]. In WT mice and PGRN^−/−^ mice, lung injury and pulmonary edema were alleviated by PGRN treatment after LPS exposure (Figures 1(a) and 1(b)). In addition, we also observed that the expression levels of proinflammatory cytokines (IL-1*β*, IL-6, IL-17A, and TNF-*α*) and chemokines (CXCL1) decreased following PGRN treatment after exposure to LPS at 24 h (Figure 1(c)). IHC images of mouse lungs showed that apoptosis and MPO-expressing neutrophils were also clearly reduced after PGRN treatment (Figures 2(a) and 2(c)). The strong evidence generally supports a strong role for PMNs in ARDS. But not all studies found the same tendency of alteration in PMN function in blood or BALF from ARDS patients. It may be due to the heterogeneous etiologies of ARDS [47]. Our study showed that with treatment with PGRN, the total number of PMNs decreased in LPS-induced ALI (Figure 2(d)). These results suggested that PGRN improves LPS-induced ALI along with uncontrolled inflammation, pulmonary edema, neutrophil aggregation, and apoptosis and injury of alveolar epithelium and vascular endothelial cells in both WT and PGRN^−/−^ mice.

Full-length PGRN has a well-known anti-inflammatory effect, but granulin (GRN), the degradation product of PGRN, is thought to have a proinflammatory role [14, 48, 49]. Moreover, in some specific disease states (such as obesity and insulin-resistant diabetes), PGRN has a proinflammatory effect. Therefore, the exact effect of PGRN varies depending on the stages or components involved in inflammation [17] and the pathological context [50]. It has been reported that PGRN is a ligand of TNFR [18], but existing research suggests that the anti-inflammatory activity of PGRN in ARDS is not mediated solely by the PGRN/TNFR2 interaction [19]. ALI has been described as a proinflammatory pathology mediated by cells of the innate immune system [34, 51]. Some independent evidence indicates that there are interactions of PGRN with TNFR in various cell types, including human lymphocytes [25, 52–54]. Tregs, a functional subpopulation of T lymphocytes, are of great importance for immune homeostasis and self-tolerance [55]. They play a core role in the alleviation or treatment of ARDS in that they orchestrate a complex series of therapeutic events [34]. Most importantly, Tregs have been shown to participate in ALI as a suppressive mediator [56]. Study showed that CD4^+^CD25^+^FoxP3^+^ Treg is a critical effector to protect against transfusion-related acute lung injury (TRALI). Treg depletion *in vivo* resulted in aggravated antibody-mediated acute lung injury within 90 minutes [37]. Some evidence has shown that PGRN regulates Tregs, including activated T lymphocytes, to enhance their conversion into iTregs [50] and promotes TNF-induced proliferation of suppressive mouse CD4^+^Foxp3^+^ regulatory T cells [53]. PGRN preferentially promotes the proliferation of Tregs driven by TNF without T cell receptor stimulation [52]. In this study, the percentages of Tregs in both PBMCs and splenic MNCs increased significantly after PGRN treatment (Figures 3(a) and 3(b)). Thus, the immunosuppressive activity of PGRN was associated with its effect on Tregs in ALI.

IL-10 is a critical mediator of PGRN-mediated anti-inflammation [25], and plasma IL-10 is associated with the development of ARDS [57]. The anti-inflammatory properties of IL-10 may regulate the inflammatory response of the lungs, improve oxygenation, and inhibit oxidative stress [58]. The immunological mechanism that underlies the PGRN-mediated therapeutic effects in inflammatory ARDS, in particular, the molecular regulation of PGRN-mediated IL-10 production, is not clear. In this study, the expression of anti-inflammatory IL-10 in plasma increased by PGRN treatment after LPS (Figure 1(c)), while the expression of IL-10 in lung decreased (Figure 2(b)). In ALI, IL-10 is rapidly produced and significantly contributes to immunopathogenesis [34], not only by repressing the production of proinflammatory cytokines but also by restraining the activity of neutrophils [24]. The interaction between Tregs and other cells affected by cytokines from Tregs is important in this research. IL-10 is an anti-inflammatory cytokine. It has been confirmed to be associated with ARDS in patients [57]. Tregs can produce IL-10 to suppress uncontrolled immune responses and thus to protect the host [22]. IL-10 expression is regulated by Tregs in the progress of ALI, and sometimes, the effect of IL-10 may inhibit TNF-*α* production [34]. Studies showed that CD4^+^ Tregs are protective in antibody-mediated acute lung injury (TRALI) and that protection is associated with increased IL-10 levels, but when CD4^+^ Tregs are depleted, IL-10 levels are low and acute lung injury occurs [37]. In contrast, alveolar CD4^+^ CD25^+^ FoxP3^+^ T regulatory cells were reported to be increased in ARDS patients, with a concomitant correlation with increased IL-10 levels [59]. The relationship between IL-10 and Tregs differs from the types of ARDS. It is important to establish an appropriate model, including specific categories (such as TRALI), to illuminate the role of T regulatory cells and IL-10 in ARDS more firmly. However, the anti-inflammatory effects of PGRN on the regulation of IL-10 expression may be mediated by other ways. Specifically, because of the inflammatory inhibition of PGRN, the overall inflammatory response is reduced, namely, proinflammatory factors (IL-1*β*, IL-6, IL-17A, TNF-*α*, and CXCL1). Hence, the expression of the anti-inflammatory factor IL-10 subsequently reduced as a corresponding response (Figure 2(b)). In addition, the results showed that the expression of IL-10 was upregulated significantly in LPS-induced ALI WT mice after being treated with PGRN. However, in the LPS-induced ALI PGRN^−/−^ mice, the upregulation of IL-10 was not obvious after PGRN treatment (Figure 1(c)). It may be related to PGRN reducing the overall intensity of the inflammatory response. So, it seems that the feedback loop of IL-10 and immune cells is extremely complex. M1 aggravates lung injury by releasing various proinflammatory mediators, whereas M2 alleviates lung injury by eliminating apoptotic cells and participates in lung tissue repair in ARDS [60, 61]. Our experiments showed that PGRN reduced M1 and increased M2 in the lungs of the LPS-induced mouse ALI model (Figure 4). *In vitro*, IL-10 can increase the polarization of M2 macrophages and reduce the polarization of M1 macrophages (Figure 5(b)). The anti-inflammatory effect of IL-10 is mediated by metabolic reprogramming of macrophages [62], which is a key event in the inflammatory response, including inhibition of mTOR signalling and inflammasome activation. Study showed that Treg-derived IL-10 is the main factor that could affect macrophage polarization [63]. This may be another IL-10 immune modulation mechanism of PGRN in ARDS. Furthermore, CD4^+^CD25^+^ Tregs can promote the differentiation of M2 macrophages *in vivo* by transferring directly into the peritoneal cavity of severe combined immunodeficiency mice [64]. More importantly, PGRN signalling is TNFR2-dependent, and the TNFR2 signalling pathway is required for the expansion and activation of Tregs and the production of IL-10 [25]. These studies imply that Tregs are the predominant new source of IL-10 in response to PGRN, IL-10 expression is regulated by Tregs, and the effect of IL-10 may inhibit TNF-*α* production [34]. Therefore, we used recombinant PGRN to activate the differentiation of Tregs from naïve CD4^+^ T cells and to detect the expression of IL-10 *in vitro*. Interestingly, after treatment with recombinant PGRN, the percentage of CD4^+^CD25^+^Foxp3^+^ Tregs increased markedly (Figure 5). However, the detection of IL-10 by ELISA was difficult, and the value of the control group without PGRN intervention was below the limit of detection, which was in accordance with the views that Tregs usually do not produce detectable amounts of IL-10 ex vivo under physiological conditions [65], unless isolated from the gut [66] or following antigenic stimulation by antigen-presenting cells [67]; moreover, the properties of regulatory T cells sometimes cannot be predicted from *in vitro* studies [68]. Such evidence suggests that Tregs were the predominant source of IL-10 in response to PGRN. Thus, the protective effect of PGRN on ARDS may involve regulating naïve CD4^+^ T cells to differentiate into Tregs.

In summary, the protective effects of PGRN on ARDS involve the regulation of inflammatory responses through cytokines/chemokines secreted by Tregs. This study shows that PGRN promotes the differentiation of naïve CD4^+^ T cells mainly into CD4^+^CD25^+^FOXP3^+^ Tregs. Because PGRN signalling dependence on TNFR2 has been confirmed, we did not study the signalling pathway and activation of Tregs. We instead focused on the influence of PGRN on the differentiation of Tregs and its subsequent effects. Our results suggested that Tregs are a potential immune cell target for the protective effects of PGRN on LPS-induced ALI. In addition, the efficacy of PGRN treatment in IL-10 immune modulation in the ALI model indicates that PGRN is a promising approach for the treatment of acute inflammatory diseases.

## 5. Conclusions

PGRN can reduce the severity of ALI and uncontrolled inflammation in part by promoting the differentiation of naïve CD4^+^ T cells into CD4^+^CD25^+^Foxp3^+^ Tregs. Regulating IL-10 immune modulation is an important part of the anti-inflammatory effect of PGRN in ALI. The mechanism is in part mediated by the regulation of Treg differentiation.

## Data Availability

All raw data used to support the findings of this study are available from the corresponding author upon request.
